# Invasively measured and estimated central blood pressure using the oscillometric algorithm Antares in patients with and without obesity

**DOI:** 10.1371/journal.pone.0294075

**Published:** 2023-12-14

**Authors:** Alexander Stäuber, Marcus Dörr, Cornelia Piper, Marco Köster, Harald Lapp, Stefan Richter, Marc-Alexander Ohlow, Siegfried Eckert, Matthias Wilhelm Hoppe, Michael Thomas Coll Barroso, Johannes Baulmann

**Affiliations:** 1 Department of Movement and Training Science, Leipzig University, Leipzig, Germany; 2 Department of Internal Medicine B, University Medicine Greifswald, Greifswald, Germany; 3 Clinic for General and Interventional Cardiology/Angiology, Heart and Diabetes Center North Rhine-Westphalia, Bad Oeynhausen, Germany; 4 Department of Cardiology, Zentralklinik Bad Berka GmbH, Bad Berka, Germany; 5 Department of Cardiology, SRH Klinikum Burgenlandkreis GmbH, Naumburg, Germany; 6 Department of Cardiology, SRH Wald-Klinikum GmbH, Gera, Germany; 7 Department of Training Science, Philipps-Universität Marburg, Marburg, Germany; 8 Department of Internal Medicine, Fliedner University of Applied Sciences, Düsseldorf, Germany; 9 Praxis Dres. Gille/Baulmann, Rheinbach, Germany; University of Perugia, ITALY

## Abstract

**Background:**

Obesity is a global health concern and risk factor for cardiovascular disease. The assessment of central blood pressure (cBP) has been shown to improve prediction of cardiovascular events. However, few studies have investigated the impact of obesity on cBP in adults, and invasive data on this issue are lacking. This study aimed to evaluate cBP differences between patients with and without obesity, identify cBP determinants, and evaluate the accuracy of the algorithm Antares for non-invasive cBP estimation.

**Methods:**

A total of 190 patients (25% female; 39% with BMI ≥30kg/m^2^; age: 67±12 years) undergoing elective cardiac catheterization were included. cBP was measured invasively and simultaneously estimated non-invasively using the custo screen 400 device with integrated Antares algorithm.

**Results:**

No significant cBP differences were found between obese and non-obese patients. However, females, especially those with obesity, had higher systolic cBP compared to males (P<0.05). Multiple regression analysis showed that brachial mean arterial pressure, pulse pressure, BMI, and heart rate predicted cBP significantly (adjusted R^2^ = 0.82, P<0.001). Estimated cBP correlated strongly with invasive cBP for systolic, mean arterial, and diastolic cBP (r = 0.74–0.93, P<0.001) and demonstrated excellent accuracy (mean difference <5 and SD <8 mmHg).

**Conclusions:**

This study discovered no significant difference in cBP between obese and non-obese patients. However, it revealed higher cBP values in women, especially those with obesity, which requires further investigation. Additionally, the study highlights Antares’ effectiveness in non-invasively determining cBP in obese individuals. This could improve the diagnosis and treatment of hypertension in this special patient population.

## Introduction

The prevalence of obesity has reached pandemic proportions. In fact, about 25% of adults in Western countries are obese today. This has led to an increase in the risk of cardiometabolic comorbidities [1]. According to the Non-Communicable Disease Collaboration analyses, global rates of obesity (i.e., body mass index; BMI ≥30 kg/m²) range between 11% and 15%, with data from nearly 20 million adults showing that the global prevalence of obesity doubled between 1975 and 2014 [2]. While a very small proportion of obesity cases are due to monogenic changes, the increasing prevalence of general (multifactorial) obesity over the past 50 years is most likely due to a complex interaction of environmental changes (including high-calorie food intake, physical inactivity) and individual genetic predisposition [3]. Obesity is associated with higher cardiovascular risk and earlier onset of cardiovascular morbidity. Also, it is associated with activation of the sympathetic nervous system and the renin-angiotensin system, which contributes to the development of hypertension [4, 5]. It has been shown that central (aortic) blood pressure (cBP), in addition to the information yielded by conventional brachial blood pressure (BP), provides information on end-organ damage, may predict vascular events better than brachial BP, and is closely related to cardiovascular and cerebrovascular end points [6–10]. The results of a twin study in adults also suggest a significant age-, sex-, and country-adjusted heritability of the cBP components (systolic cBP and central pulse pressure) that is higher than that observed for the corresponding brachial pressures, which may underscore the importance of cBP determination for risk stratification and hypertension management [11]. To date, there are only few studies that have examined the effects of obesity on cBP using the non-invasive technique of applanation tonometry. The findings indicate that obese have a higher cBP than non-obese individuals [12, 13]. In contrast, overweight and obese also tend to have lower cBP than lean patients, especially women [14]. In addition, an inverse association was found between BMI and cBP [14]. However, studies that have invasively examined cBP in adult obese patients have not been conducted to date. Apart from using invasive BP measurement as the gold standard, there exist other reliable non-invasive techniques to determine cBP, besides applanation tonometry [15]. In this regard, the Antares algorithm offers an alternative approach to estimating cBP by examining pulse wave characteristics, which can be incorporated into a standard oscillometric BP monitor to function as a type II pulse wave analysis (PWA) device. Oscillometric BP measurements rely on the analysis of oscillometric pulses within an inflatable cuff during its gradual deflation, starting from a pressure level above systolic BP and concluding below diastolic BP. This analytical approach involves separating and monitoring the amplitudes of these oscillations as the cuff pressure progressively decreases. The oscillation amplitude attains its maximum when the cuff pressure aligns with the mean arterial pressure (MAP). The upper boundary of the oscillometric waveform is denoted as the oscillometric waveform envelope (OMWE). Traditionally, systolic, and diastolic BP are subsequently calculated based on the characteristics of the OMWE, with each manufacturer of oscillometric BP monitors using their own proprietary algorithm [16]. The oscillometric algorithm Antares has been validated invasively in a patient population comprising of individuals with a range of cardiovascular and cardiometabolic conditions [17, 18]. However, it is not known whether Antares provides accurate non-invasive cBP values compared with the invasive method in obese patients yet.

Thus, our study aimed to examine potential differences in invasively measured cBP among patients who are normal-weight, overweight, and obese. We also aimed to identify determinant factors for cBP and to evaluate the accuracy of the Antares algorithm in this patient population.

## Materials and methods

### Study population

This study was conducted between 10/16/2017 and 2/16/2021 as part of a multicenter validation study collecting data from three different hospitals in Germany: Greifswald, Bad Oeynhausen and Bad Berka. This research received approval from three ethics committees: Landesärztekammer Thüringen (Reg.-Nr.: 36950/2018/76), Ethics Committee University Medicine Greifswald (Reg.-Nr.: BB032/17), and Ethics Committee Ruhr University Bochum (Reg.-Nr.: 2017–219). The study strictly followed the principles outlined in the Declaration of Helsinki, and all participants provided written informed consent before participating in the study. According to the data exclusion procedures, 190 patients could be included in the final data analysis (S1 Fig).

### Clinical characteristics and BP measurements

Based on the World Health Organization (WHO) classification, the BMI was categorized as follows: 18.5–24.9 kg/m^2^ for normal weight, 25.0–29.9 kg/m^2^ for overweight, and at least 30.0 kg/m^2^ for obesity [19]. Clinical information with corresponding diagnoses were taken from the patient’s medical records at the respective study centers. The diagnosis of arterial hypertension followed the definition of the 2018 ESC/ESH guideline [20]. The systolic cBP (cSBP), diastolic cBP (cDBP), and central mean arterial pressure (cMAP) were recorded by conducting simultaneous invasive (ascending aorta) and non-invasive measurements in the cardiac catheterization labs. Using the collected data, the pulse pressure (PP) was calculated for both brachial and central BP measurements by subtracting the diastolic BP from the systolic BP. The invasive BP devices used comply with the requirements of ISO 81060–2:2019 [21]. Data collection was carried out during a resting period, free from any acute hemodynamic interventions or medication changes, and without any conversations taking place. Invasive measurements were taken using fluid-filled catheters with either radial (n = 160, 84%) or femoral (n = 30, 16%) catheter access. The highest point of each accurately recorded pulse wave was utilized to determine the invasive systolic BP, while the lowest signal point was used to ascertain the invasive diastolic BP. Furthermore, the area under the curve was utilized for calculating the invasive mean arterial pressure (MAP). Simultaneously, the cuff-based non-invasive measurements were conducted on the left upper arm using the custo screen 400 device (Custo med GmbH, Ottobrunn, Germany), which incorporates the Antares algorithm (Redwave Medical GmbH, Jena, Germany) to calculate cBP. These cuff-based measurements were also used to determine brachial BP. The patent for pulse wave analysis (PWA) in oscillometric pulse waves recorded during the inflation and deflation of a cuff (patent no. DE 10 2017 117 337 B4) is held by Redwave Medical. In brief, the Antares algorithm extracts the pulsatile signal from cuff pressure during deflation, identifies individual pulse waves, and applies a weighted transform using an adaptive transfer function to each wave. Grid points are identified to calculate hemodynamic parameters like cBP, and a residual is calculated by comparing actual and expected cuff pressure. Arrhythmias and artifacts are detected based on the residual and pulse wave shape. Integrating Antares into a BP device software can make it a type II PWA device with accurate absolute cBP values independent of peripheral BP measurement. The algorithm only requires recorded pulse wave shapes and sex information from the patient. More detailed information on the measurement approach can be found elsewhere [17].

### Statistical analyses

The normal distribution of data was assessed using the Shapiro-Wilk test. The differences between the normal-weight, overweight, and obese groups were assessed based on the distribution of continuous variables using one-way analysis of variance (ANOVA) with Bonferroni post hoc test or Kruskal-Wallis test with Mann-Whitney U test as post hoc test, and the chi-square test for categorical variables. Differences between sexes were analysed using Student’s t-test. Partial correlations adjusted by sex and age were used to investigate associations between cBP, brachial BP and BMI. For the evaluation of predictive factors of invasive cBP multiple linear regression analyses were performed using the inclusion method (models 1,2) with invasive cBP as dependent variable and age, sex, BMI, heart rate, brachial SBP, MAP and BP treatment as independent variables according to the methodological approach and findings of Pichler et al. [14]. In addition, the stepwise backward elimination method (model 3) was performed with cBP as the dependent variable and age, sex, BMI, heart rate, brachial SBP, MAP, PP, BP treatment, dyslipidaemia, type 2 diabetes mellitus, chronic kidney disease, stroke, myocardial infarction, percutaneous coronary intervention (PCI), coronary artery disease, and heart failure as independent variables. This method was chosen because, on the one hand, many independent variables can be considered as predictors and a restriction to important variables is not possible by previous studies. Due to the stepwise backward elimination, it is least likely not to include variables that in fact have an influence (suppressor effects) [22]. Collinearity between all variables was assessed using appropriate statistical methods.

The invasively measured aortic reference cBP, along with its SD, represents the instantaneous BP variation during the estimation of cBP by Antares. According to ISO 81060–2:2019 [21], Antares’ measurement error is considered 0 mmHg when the estimated cBP falls within this range. If the calculated cBP is outside the range of invasive aortic reference BP, the adjacent range limit value of the invasive reference cBP is subtracted from the calculated cBP, representing the measurement error. This error was used to determine the mean difference between the invasive and non-invasive cBP recording in the study population. Agreement between the invasive and non-invasive cBP was evaluated using Bland-Altman plots with limits of agreement (±1.96 SD). In accordance with distribution of data the Pearson product-moment correlation coefficient was used to assess the strength of linear association between invasive and non-invasive cBP. In addition, scatter plots were created for a graphical overview. A two-sided P value of less than 0.05 was considered statistically significant in all analyses. All analyses were done using SPSS version 22 (IBM Corp, Armonk, New York, USA).

## Results

### Characteristics of the study population

The characteristics of the study population are shown in Table 1.

**Table 1:** General characteristics of the study population.

	Total	Normal-weight	Overweight	Obesity	*P* value
Patients, n	190	39	76	75	
Male/female, n (%)	143 (75) / 47 (25)	30 (77) / 9 (23)	55 (72) / 21 (28)	58 (77) / 17 (23)	0.75
Age (years)	66.7 ± 12.1 (66.0)	65.8 ± 13.0 (64.0)	67.6 ± 12.2 (68.0)	66.3 ± 11.5 (65.0)	0.69^#^
Weight (kg)	85.9 ± 16.2 (84.5)	69.0 ± 8.5 (70.0)	82.2 ± 10.4* (81.0)	98.8 ± 14.1*/** (97.0)	<0.001^#^
Height (m)	172.6 ± 9.2 (172.0)	174.1 ± 8.5 (176.0)	172.8 ± 9.4 (172.0)	171.9 ± 9.3 (172.0)	0.49^#^
BMI (kg/m^2^)	28.8 ± 4.6 (28.7)	22.7 ± 1.8 (23.1)	27.4 ± 1.5* (27.3)	33.3 ± 3.1*/** (32.3)	<0.001^§^
**Clinical characteristics**					
Hypertension	166 (87%)	32 (82%)	64 (84%)	70 (93%)	0.17
Dyslipidaemia	92 (48%)	17 (44%)	42 (55%)	33 (44%)	0.29
Type 2 diabetes mellitus	73 (38%)	8 (21%)	28 (37%)*	37 (49.5%)*/**	0.01
Chronic kidney disease	26 (14%)	6 (15%)	9 (12%)	3 (15%)	0.82
Stroke	14 (7%)	2 (5%)	9 (12%)	3 (4%)	0.14
Myocardial infarction	54 (28%)	12 (31%)	19 (25%)	23 (31%)	0.74
Patients undergoing PCI	101 (53%)	20 (51%)	33 (43%)	48 (64%)**	0.04
Heart failure	37 (20%)	9 (23%)	16 (21%)	12 (16%)	0.59
Coronary heart disease	119 (63%)	26 (67%)	46 (60%)	47 (63%)	0.89
Heart rate (1/min)	66.6 ± 11.8 (65.0)	68.5 ± 13.4 (67.0)	64.7 ± 11.3 (63.5)	67.5 ± 11.3 (66.0)	0.19^§^

Numeric data are mean ± SD (Median). Normal weight, BMI: 18.5–24.9 kg/m^2^; Overweight, BMI: 25.0–29.9 kg/m^2^; Obesity, BMI: ≥30 kg/m^2^. BMI, body mass index; PCI, percutaneous coronary intervention; P value:

^#^one-way analysis of variance with Bonferroni test for post hoc analysis and

^§^Kruskal-Wallis test with Mann-Whitney U test for post hoc analysis for numeric variables and chi-square test for categoric variables.

*P<0.05 with normal-weight group

**P<0.05 with overweight group

All 190 included patients were Caucasian, of whom cardiac catheter access was radial in 160 cases (84%) and femoral in 30 cases (16%). Eleven patients (6%) were younger than 50 years, 107 patients (56%) were between 50 and 70 years, and 72 patients (38%) were older than 70 years. 127 patients (67%) were within a resting heart rate of 60–100 beats per minute (67%). 38 patients reported a history of smoking (20%). According to BMI, 39% of the study population was obese, 40% had overweight and 21% normal-weight. The proportion of female patients was 25% in the total sample as well as within each of the three BMI groups. Compared to the overweight and normal weight groups, the obese group showed the highest prevalence of type 2 diabetes, with the normal weight group exhibiting the lowest prevalence. Additionally, the obese group had a significantly higher number of cases undergoing PCI than the overweight group. No further significant differences were observed between the groups.

### Comparison of central and brachial BP between patients with normal-weight, overweight and obesity

The central and brachial BP values for the total population and BMI-stratified groups are displayed in Table 2.

**Table 2:** Invasive and non-invasive oscillometric central (aortic) BP and oscillometric brachial BP in normal-weight, overweight and obese patients.

	Total	Normal-weight	Overweight	Obesity	*P* value
Patients, n	190	39	76	75	
**Invasive central (aortic) BP**					
SBP (mmHg)	133.3 ± 22.5 (132.6)	132.4 ± 25.6 (132.0)	136.1 ± 23.4 (137.2)	130.7 ± 19.3 (129.9)	0.25^#^
DBP (mmHg)	68.4 ± 10.6 (67.9)	67.1 ± 12.2 (67.9)	69.7 ± 11.2 (68.4)	67.8 ± 8.8 (67.5)	0.44^#^
MAP (mmHg)	94.6 ± 12.8 (94.9)	93.7 ± 15.8 (92.9)	96.4 ± 13.3 (96.3)	93.3 ± 10.3 (93.0)	0.29^#^
PP (mmHg)	64.9 ± 20.7 (61.3)	65.3 ± 20.9 (64.6)	66.5 ± 21.7 (64.4)	62.8 ± 19.3 (59.4)	0.43^§^
**Non-invasive oscillometric central (aortic) BP**					
SBP (mmHg)	132.5 ± 22.5 (132.2)	130.5 ± 23.5 (129.1)	135.8 ± 24.1 (133.0)	131.8 ± 20.6 (130.7)	0.56^#^
DBP (mmHg)	72.2 ± 11.1 (71.7)	71.8 ± 11.6 (70.8)	73.7 ± 12.3 (71.9)	72.2 ± 10.8 (71.4)	0.89^#^
MAP (mmHg)	94.6 ± 12.9 (95.1)	93.6 ± 14.3 (90.8)	96.8 ± 14.0 (96.1)	94.1 ± 11.9 (95.0)	0.53^#^
PP (mmHg)	60.4 ± 20.6 (56.3)	58.7 ± 20.4 (57.8)	62.1 ± 21.8 (60.7)	59.6 ± 19.2 (54.5)	0.66^§^
**Oscillometric brachial BP**					
SBP (mmHg)	139.5 ± 20.9 (138.5)	136.7 ± 22.1 (138.0)	142.3 ± 22.8 (138.5)	140.3 ± 19.3 (140.0)	0.63^#^
DBP (mmHg)	80.7 ± 10.0 (80.0)	79.4 ± 10.8 (79.0)	82.0 ± 11.3 (80.0)	81.2 ± 9.6 (81.0)	0.68^#^
MAP (mmHg)	100.2 ± 14.2 (100.0)	97.6 ± 15.2 (97.0)	102.3 ± 15.6 (101.0)	100.7 ± 13.0 (101.0)	0.44^#^
PP (mmHg)	58.9 ± 16.3 (56.0)	57.3 ± 16.5 (59.0)	60.3 ± 17.6 (57.0)	59.1 ± 15.2 (55.0)	0.87^§^

Data are mean ± SD (Median). Normal weight, BMI: 18.5–24.9 kg/m^2^; Overweight, BMI: 25.0–29.9 kg/m^2^; Obesity, BMI: ≥30 kg/m^2^. BMI, body mass index; BP, blood pressure; SBP, systolic blood pressure; DBP, diastolic blood pressure; MAP, mean arterial pressure; PP, pulse pressure; P value:

^#^one-way analysis of variance and

^§^Kruskal-Wallis test for numeric variables

There were no significant differences between the groups. Also, sex-specific analysis showed no statistically significant differences between the BMI groups for brachial and central BP in women and men (S1 and S2 Tables). Comparing the sexes between the total group and BMI subgroups revealed significant differences, particularly in the cBP of overweight and obese patients (P<0.05). The results indicated that overweight female patients had significant lower cDBP and higher central pulse pressure (cPP) than their male counterparts (P<0.05). Obese female patients showed significant higher cSBP and cPP than males (S1 and S2 Tables). S3 and S4 Tables summarize the partial correlations among BMI, brachial BP, and cBP, controlled for sex and age, which show no significant correlation between BMI and BP values.

### Determinants of cSBP

The multiple linear regression analysis results are presented in Table 3.

**Table 3:** Multiple linear regression analysis of determinant factors of invasive central (aortic) systolic BP.

Variable	cSBP			
	B [CI95%]	Beta	*P* value	Adjusted R^2^
**Model 1**				0.79
Age (years)	0.11 (-0.02/0.23)	0.06	0.09	
Sex (male vs. female)	-3.98 (-7.45/-0.52)	-0.08	0.03	
BMI (kg/m^2^)	-0.56 (-0.88/-0.24)	-0.12	<0.001	
Brachial SBP (mmHg)	0.94 (0.87/1.02)	0.88	<0.001	
BP treatment (yes vs. no)	1.23 (-3.56/6.02)	0.02	0.61	
**Model 2**				0.74
Age (years)	0.25 (0.11/0.39)	0.14	<0.001	
Sex (male vs. female)	-5.25 (-9.11/-1.38)	-0.10	0.01	
BMI (kg/m^2^)	-0.62 (-0.98/-0.26)	-0.13	<0.001	
Brachial MAP (mmHg)	1.34 (1.22/1.46)	0.84	<0.001	
Heart rate (1/min)	-0.28 (-0.42/-0.14)	-0.15	<0.001	
BP treatment (yes vs. no)	2.44 (-2.89/7.78)	0.04	0.37	
**Model 3**				0.82
BMI (kg/m^2^)	-0.52 (-0.82/-0.22)	-0.11	<0.001	
Brachial MAP (mmHg)	0.77 (0.62/0.92)	0.48	<0.001	
Brachial PP (mmHg)	0.68 (0.55/0.81)	0.49	<0.001	
Heart rate (1/min)	-0.14 (-0.26/-0.02)	-0.07	0.03	

BMI, body mass index; BP, blood pressure; cSBP, central (aortic) systolic blood pressure; DBP, diastolic blood pressure; MAP, mean arterial pressure; PP, pulse pressure

Models 1 and 2 revealed significant negative associations between cSBP and both BMI and male sex (P<0.05). BP treatment did not prove to be a significant predictor, while brachial SBP (model 1) or brachial MAP, age and heart rate (model 2) showed significant associations with cSBP (P<0.001). Model 3 exhibited the highest adjusted R-squared value, wherein brachial PP and MAP were the most prominent predictors based on the standardized regression coefficient beta, followed by BMI and heart rate with negative associations.

### Accuracy of Antares cBP

**Fig 1:**
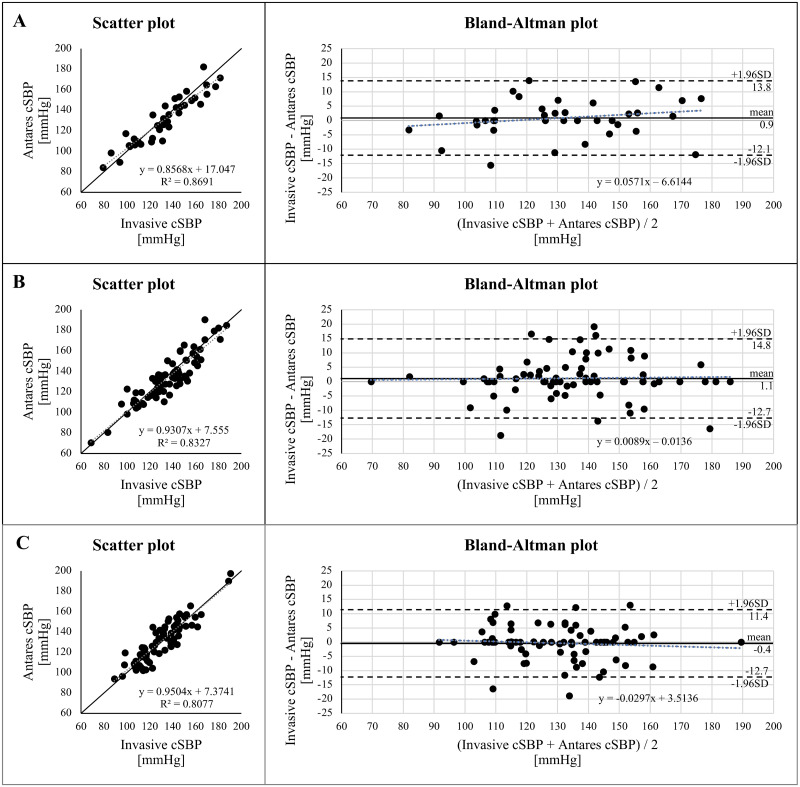
Relationship and comparison of non-invasive central (aortic) systolic blood pressure (cSBP) calculated with Antares compared with invasive cSBP in normal-weight (A), overweight (B) and obese patients (C). Grey dotted line: linear regression line. Black line: identity line. R2, coefficient of determination. Bland-Altman plot for invasive cSBP and Antares cSBP with the representation of mean difference (black line) and limits of agreement (black dashed line), from ±1.96SD. Mean difference ± SD: 0.9 ± 6.6 mmHg (A), 1.1 ± 7.0 mmHg (B), -0.4 ± 6.0 mmHg (C).

A significant and strong correlation was found between non-invasively calculated cBP using Antares and invasively measured cBP for cSBP (r = 0.89–0.93, P<0.001), cMAP (r = 0.84–0.92, P<0.001), and cDBP (r = 0.74–0.87, P<0.001) in patients with normal weight, overweight, and obesity. The Bland-Altman plots in Fig 1 display the mean difference and SD for cSBP in different BMI groups, indicating good limits of agreement.

**Fig 2:**
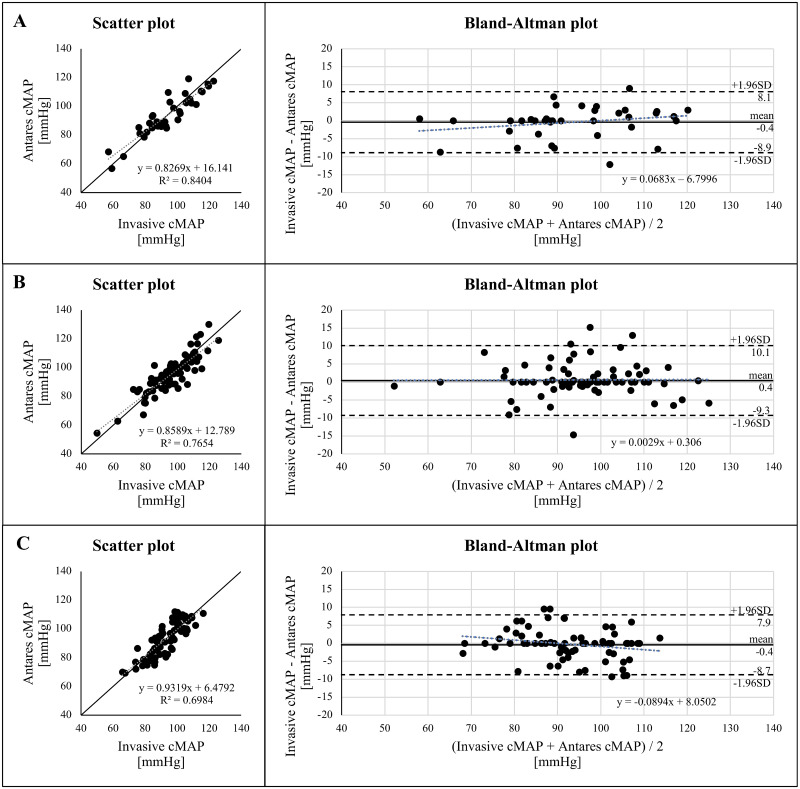
Relationship and comparison of non-invasive central (aortic) mean arterial blood pressure (cMAP) calculated with Antares compared with invasive cMAP in normal-weight (A), overweight (B) and obese patients (C). Grey dotted line: linear regression line. Black line: identity line. R2, coefficient of determination. Bland-Altman plot for invasive cMAP and Antares cMAP with the representation of mean difference (black line) and limits of agreement (black dashed line), from ±1.96SD. Mean difference ± SD: -0.4 ± 4.3 mmHg (A), 0.4 ± 4.9 mmHg (B), -0.4 ± 4.2 mmHg (C).

**Fig 3:**
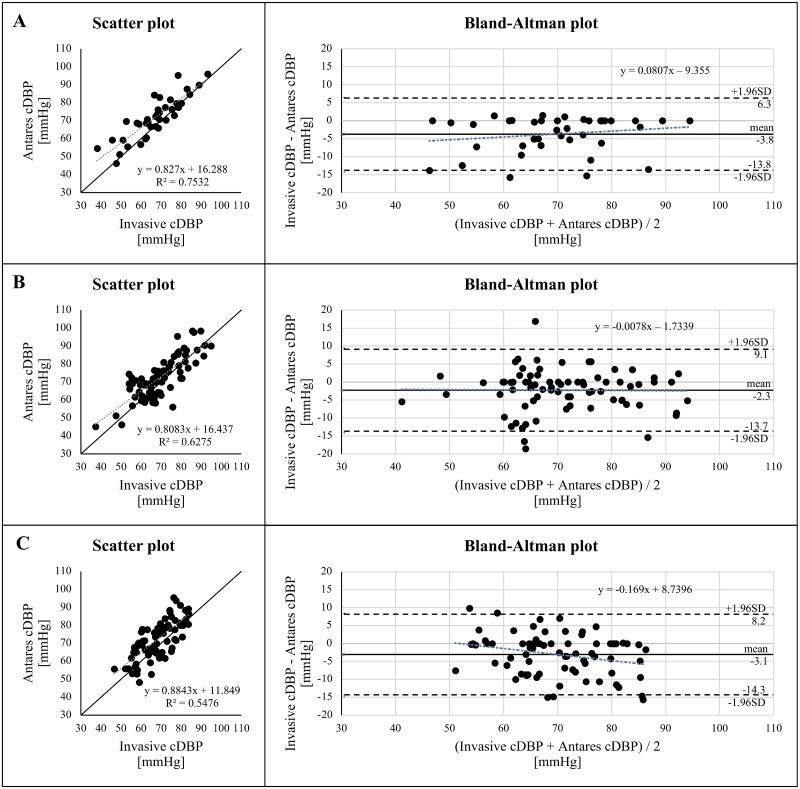
Relationship and comparison of non-invasive central (aortic) diastolic blood pressure (cDBP) calculated with Antares compared with invasive cDBP in normal-weight (A), overweight (B) and obese patients (C). Grey dotted line: linear regression line. Black line: identity line. R2, coefficient of determination. Bland-Altman plot for invasive cDBP and Antares cDBP with the representation of mean difference (black line) and limits of agreement (black dashed line), from ±1.96SD. Mean difference ± SD: -3.8 ± 5.1 mmHg (A), -2.3 ± 5.8 mmHg (B), -3.1 ± 5.7 mmHg (C).

Additionally, Figs 2 and 3 show the data for cMAP and cDBP, respectively. No significant overestimations or underestimations have been found for cSBP, cMAP, or cDBP.

## Discussion

The present study is the first to investigate the impact of overweight and obesity on invasively measured cBP. The findings revealed no significant difference of cBP in male and female patients with normal weight, overweight, and obesity. However, obese female patients showed significant higher cSBP compared to male patients. Moreover, our study demonstrated a significant negative association between BMI and invasive cSBP using various models in the multiple linear regression analysis. Within model 3 with the highest goodness-of-fit measure (R^2^ = 0.82), BMI, brachial MAP, brachial PP, and heart rate were identified as the most significant predictors of invasive measured cSBP. Our data also confirmed the validity of the Antares algorithm in accurately determining cBP in all BMI groups.

Previous research on obesity and cSBP in adult patients is limited to a few studies that used exclusively non-invasive techniques for measuring cBP, yielding inconclusive results [12–14, 23–25]. Westerbacka et al. [12] and Chao et al. [13] have shown, that obese adults have significantly higher cBP than normal-weight adults. In contrast, Pichler et al. [14] found that normotensive and hypertensive adult female patients with overweight and obesity tend to have lower cBP compared to lean female patients. But no differences have been found for lean, overweight, and obese male patients [14]. Our study could not demonstrate statistically significant cBP differences between BMI groups for either the total sample or sex-stratified sub analysis. Even though obese patients had a higher prevalence of type 2 diabetes and a larger number of patients undergoing PCI, the clinical characteristics between our groups were otherwise similar. This suggests that BMI may have a minor impact on cBP in adult patients, even though multiple regression analysis showed a negative association that is consistent with findings of Pichler et al. [14]. Paradoxically, this negative association suggests a potential cBP-lowering influence of BMI.

The difference in the previously reported and our study results could be due to several factors. First, in contrast to the study population of Westerbacka et al. [12], Chao et al. [13], and Pichler et al. [14], patients in our study were, on average, older with higher cardiovascular disease burden throughout all BMI groups, making the study results not directly comparable. In addition, the study by Chao et al. [13] was conducted in an Asian population with different cut-off values for overweight (24 kg/m^2^) and obesity (28 kg/m^2^). Consequently, the corresponding results might not be applicable to other ethnic groups like ours. Third, cBP was determined non-invasively by applanation tonometry on the radial artery in these studies rather than invasively and oscillometric as in our study. Since it is not known how exactly these non-invasive (indirect) measurement methods perform in overweight and obese patients compared with the invasive gold standard, systematic biases in the measurement results cannot be excluded. However, in analogy to the results of Pichler et al. [14], our study also found higher cSBP in female patients compared with male patients, especially in obese women. These results may be attributed to sex differences in the mechanisms of arterial stiffening during aging, hypertension, and obesity [26]. Furthermore, variations in circulation between men and women, including smaller physiological features (e.g., shorter arterial tree length and diameter), faster heart rate, and levels of estrogenic hormones in women [27], may have also contributed to differences in cSBP. Nevertheless, obesity directly contributes to cardiovascular risk factors, including dyslipidaemia, type 2 diabetes, and hypertension, and leads to the development of cardiovascular disease and cardiovascular mortality independently of other cardiovascular risk factors [28]. Based on an analysis of the International Database of Central Arterial Properties for Risk Stratification (IDCARS) to establish an outcome-based threshold for cSBP that takes into account both fatal and nonfatal cardiovascular end points [29], we note that the patients included in our study fell on average into the stage-1 (120–129 mmHg) and stage-2 hypertension (130–149 mmHg) categories, which in turn were associated with a hazard ratio of 1.90–2.44 for the 5-year risk in the occurrence of primary cardiovascular end points. In addition, female patients showed a cSBP in the range of stage 2 hypertension in all BMI groups. The IDCARS-data demonstrated that controlling central hypertension is crucial, as it increases the risk of cardiovascular and cerebrovascular disease, regardless of the individuals brachial BP status [29]. However, a single type-1 cBP monitoring device (SphygmoCor) was used for the non-invasive assessment of the cBP within IDCARS. In this context, a type I-device provide an estimation of cBP in relation to the measured brachial BP. This means that it can measure the pressure difference between central and peripheral sites with relative accuracy [30]. In contrast, a type II-device would give estimate of the intra-arterial “true” cBP (i.e., relatively accurate absolute cBP value despite inaccuracy at the peripheral site) [30].

It is well known that accurate brachial BP measurement can be difficult in obese individuals because the upper arms are often short, large, and conical, which increases the likelihood of inaccurate BP measurement [31]. Regardless, cBP determined from oscillometric brachial pulse waves proved to be very accurate in the present study. Although there was a decrease in correlation for invasive and estimated cSBP, cMAP and cDBP values with increasing BMI, the correlations in the obese group remained very high especially for cSBP (r = 0.89, P<0.001) except for cDBP (r = 0.74, P<0.001). However, the mean differences and the corresponding SD did not deteriorate with increasing BMI. Our study revealed that the Antares Algorithm has the capability to transform a standard automated oscillometric BP device into a type II-device, enabling the non-invasive assessment of cBP in patients of varying weight categories, including normal-weight, overweight, and obesity. Our findings demonstrate that Antares satisfies the validation criteria for accuracy outlined in 2017 ARTERY Society Task Force consensus statement on protocol standardization for validation of non-invasive cBP devices [30] and ANSI/AAMI/ISO 81060–2:2019 [21], with a mean difference of ≤5 mmHg and an experimental SD of ≤8 mmHg. Therefore, Antares has the potential to integrate cBP measurements into regular clinical practice for managing disease and risk in obese patients.

Our study has several limitations that should be acknowledged. Firstly, the cross-sectional design of the study does not allow for the determination of causal mechanisms of cBP development, which means that our findings cannot infer any possible adaptation in obesity. Additionally, the age distribution of our patient population was skewed towards older adults, with only a small number of patients younger than 50 years of age. This is because younger people rarely require cardiac catheterization. Another limitation was the small proportion of female participants, which represented only 25% of the study population. Another constrained was the limited number of subjects within the normal-weight category. Moreover, we did not gather additional indicators of central obesity, such as waist circumference, waist-to-hip ratio, or waist-to-height ratio, nor did we obtain measurements of body fat distribution through techniques such as dual-energy X-ray absorptiometry (DXA) or bioelectrical impedance analysis (BIA) to conduct a more thorough analysis. Subsequent investigations should explore whether central obesity parameters have a correlation with cBP. Additionally, the current study claims not to be a complete validation study in accordance with ARTERY [30] or ANSI/AAMI/ISO 81060–2:2019 protocol [21], as the necessary sample size and gender distribution were not provided. Nevertheless, it should be acknowledged that all other requirements were fulfilled.

## Conclusions

To summarize, this study is the first to examine how overweight and obesity affect invasively measured cBP in adult male and female patients. The results indicate that there is no significant difference in cBP between normal-weight, overweight, and obese patients. However, there are gender differences, with women having a higher cSBP, particularly if their BMI is over 30 kg/m^2^. The study also revealed a negative association between BMI and invasive cSBP, which warrants further investigation. The validity of the Antares algorithm in accurately determining cBP in patients of different weight categories was also confirmed. These findings enhance our understanding of the connection between BMI and cBP and suggest the possibility of using a conventional automated oscillometric BP device for non-invasive cBP assessment, which could improve hypertension diagnosis and treatment in overweight and obese individuals.

## Supporting information

S1 Table Invasive and non-invasive oscillometric central (aortic) BP and oscillometric brachial BP in normal-weight, overweight and obese male patients. Data are mean ± SD (Median). Normal weight, BMI: 18.5–24.9 kg/m^2^; Overweight, BMI: 25.0–29.9 kg/m^2^; Obesity, BMI: ≥30 kg/m^2^. BMI, body mass index; BP, blood pressure; SBP, systolic blood pressure; DBP, diastolic blood pressure; MAP, mean arterial pressure; PP, pulse pressure; P value: #one-way analysis of variance, §Kruskal-Wallis test. Sex comparison between the total group and the groups with the same BMI was performed using Student’s t-test. *P<0.05, comparison with female group (S2 Table); **P<0.01, comparison with female group (S2 Table); ***P<0.001, comparison with female group (S2 Table).
(DOCX)

S2 Table Invasive and non-invasive oscillometric central (aortic) BP and oscillometric brachial BP in normal-weight, overweight and obese female patients. Data are mean ± SD (Median). Normal weight, BMI: 18.5–24.9 kg/m^2^; Overweight, BMI: 25.0–29.9 kg/m^2^; Obesity, BMI: ≥30 kg/m^2^. BMI, body mass index; BP, blood pressure; SBP, systolic blood pressure; DBP, diastolic blood pressure; MAP, mean arterial pressure; PP, pulse pressure; P value: #one-way analysis of variance. Sex comparison between the total group and the groups with the same BMI was performed using Student’s t-test. *P<0.05, comparison with male group (S1 Table); **P<0.01, comparison with male group (S1 Table); ***P<0.001, comparison with male group (S1 Table).
(DOCX)

S3 Table Partial correlation coefficients between invasive central (aortic) BP variables and brachial BP variables adjusted for age and sex. BMI, Body Mass Index; BP, blood pressure; bSBP, brachial systolic blood pressure; bMAP, brachial mean arterial pressure; bDBP, brachial diastolic blood pressure; bPP, brachial pulse pressure; cSBP, central systolic blood pressure; cMAP, central mean arterial pressure; cDBP, central diastolic blood pressure; cPP, central pulse pressure. *P<0.05; **P<0.001.
(DOCX)

S4 Table Partial correlation coefficients between non-invasive central (aortic) BP variables and brachial BP variables adjusted for age and sex. BMI, Body Mass Index; bSBP, brachial systolic blood pressure; bMAP, brachial mean arterial pressure; bDBP, brachial diastolic blood pressure; bPP, brachial pulse pressure; cSBP, central systolic blood pressure; cMAP, central mean arterial pressure; cDBP, central diastolic blood pressure; cPP, central pulse pressure. **P<0.001.
(DOCX)

S1 Fig Patient exclusion flowchart. BMI, body mass index; BP, blood pressure; SD, standard deviation.
(TIF)

S1 Dataset (XLSX)
