# Supplementary material for: Invasively measured and estimated central blood pressure using the oscillometric algorithm Antares in patients with and without obesity
Source: PLoS One. 2023 Dec 14;18(12):e0294075. doi: 10.1371/journal.pone.0294075 (PMC10721029; doi:10.1371/journal.pone.0294075)
Supplement: S1 Table — Data are mean ± SD (Median). Normal weight, BMI: 18.5–24.9 kg/m2; Overweight, BMI: 25.0–29.9 kg/m2; Obesity, BMI: ≥30 kg/m2. BMI, body mass index; BP, blood pressure; SBP, systolic blood pressure; DBP, diastolic blood pressure; MAP, mean arterial pressure; PP, pulse pressure; P value: #one-way analysis of variance, §Kruskal-Wallis test. Sex comparison between the total group and the groups with the same BMI was performed using Student’s t-test. *P<0.05, comparison with female group (S2 Table); **P<0.01, comparison with female group (S2 Table); ***P<0.001, comparison with female group (S2 Table). (DOCX) [file pone.0294075.s001.docx]

**S1 Table. Invasive and non-invasive oscillometric central (aortic) BP and oscillometric brachial BP in normal-weight, overweight and obese male patients**

|  | **Total** | **Normal-weight** | **Overweight** | **Obesity** | ***P* value** |
| --- | --- | --- | --- | --- | --- |
| Patients, n | 143 | 30 | 55 | 58 |  |
| **Invasive central (aortic) BP** | | | | |  |
| SBP (mmHg) | 131.3 ± 21.6*  (130.6) | 130.4 ± 26.7  (131.6) | 135.5 ± 21.3  (133.5) | 127.8 ± 18.4*  (125.4) | 0.16^#^ |
| DBP (mmHg) | 69.6 ± 10.8**  (69.0) | 67.1 ± 12.8  (67.6) | 72.1 ± 10.8**  (71.1) | 68.6 ± 9.1  (68.6) | 0.08^#^ |
| MAP (mmHg) | 94.6 ± 12.9  (95.0) | 92.7 ± 16.1  (92.6) | 97.7 ± 12.7  (98.1) | 92.6 ± 10.7  (92.2) | 0.07^#^ |
| PP (mmHg) | 61.7 ± 19.3***  (58.5) | 63.3 ± 22.6  (58.9) | 63.4 ± 19.3*  (60.2) | 59.2 ± 17.4*  (56.2) | 0.47^§^ |
| **Non-invasive oscillometric central (aortic) BP** | | | | |  |
| SBP (mmHg) | 130.7 ± 21.7  (129.1) | 128.7 ± 24.9  (124.9) | 133.9 ± 21.8  (132.1) | 128.8 ± 19.7*  (126.5) | 0.39^#^ |
| DBP (mmHg) | 73.7 ± 11.3***  (73.2) | 71.8 ± 12.2  (71.6) | 75.4 ± 11.1***  (74.8) | 73.1 ± 10.8  (73.6) | 0.32^#^ |
| MAP (mmHg) | 94.7 ± 13.0  (95.4) | 92.5 ± 14.9  (90.1) | 97.3 ± 12.5  (96.4) | 93.4 ± 12.3  (95.0) | 0.16^#^ |
| PP (mmHg) | 57.0 ± 18.7***  (52.8) | 56.9 ± 21.4  (53.1) | 58.5 ± 19.7*  (54.5) | 55.7 ± 16.4***  (51.3) | 0.75^§^ |
| **Oscillometric brachial BP** | | | | | |
| SBP (mmHg) | 138.6 ± 20.8  (138.0) | 134.8 ± 23.5  (130.0) | 141.3 ± 20.7  (138.0) | 138.2 ± 19.3  (139.0) | 0.42^#^ |
| DBP (mmHg) | 81.5 ± 10.4  (81.0) | 79.1 ± 11.4  (79.5) | 82.9 ± 10.2**  (81.0) | 81.3 ± 10.0  (81.0) | 0.28^#^ |
| MAP (mmHg) | 99.7 ± 14.1  (100) | 97.1 ± 16.0  (95.5) | 101.7 ± 13.9  (100.0) | 99.1 ± 13.1  (99.5) | 0.33^#^ |
| PP (mmHg) | 57.1 ± 15.5**  (55.0) | 55.7 ± 17.7  (55.5) | 58.1 ± 15.6  (57.0) | 56.9 ± 14.5*  (54.0) | 0.77^§^ |

*Data are mean ± SD (Median). Normal weight, BMI: 18.5-24.9 kg/m^2^; Overweight, BMI: 25.0-29.9 kg/m^2^; Obesity, BMI: ≥30 kg/m^2^. BMI, body mass index; BP, blood pressure; SBP, systolic blood pressure; DBP, diastolic blood pressure; MAP, mean arterial pressure; PP, pulse pressure; P value: ^#^one-way analysis of variance, ^§^Kruskal-Wallis test. Sex comparison between the total group and the groups with the same BMI was performed using Student's t-test.*

**P<0.05, comparison with female group (Table in S2 Table)*

***P<0.01, comparison with female group (Table in S2 Table)*

****P<0.001, comparison with female group (Table in S2 Table)*
