# Supplementary material for: Invasively measured and estimated central blood pressure using the oscillometric algorithm Antares in patients with and without obesity
Source: PLoS One. 2023 Dec 14;18(12):e0294075. doi: 10.1371/journal.pone.0294075 (PMC10721029; doi:10.1371/journal.pone.0294075)
Supplement: S2 Table — Data are mean ± SD (Median). Normal weight, BMI: 18.5–24.9 kg/m2; Overweight, BMI: 25.0–29.9 kg/m2; Obesity, BMI: ≥30 kg/m2. BMI, body mass index; BP, blood pressure; SBP, systolic blood pressure; DBP, diastolic blood pressure; MAP, mean arterial pressure; PP, pulse pressure; P value: #one-way analysis of variance. Sex comparison between the total group and the groups with the same BMI was performed using Student’s t-test. *P<0.05, comparison with male group (S1 Table); **P<0.01, comparison with male group (S1 Table); ***P<0.001, comparison with male group (S1 Table). (DOCX) [file pone.0294075.s002.docx]

**S2 Table. Invasive and non-invasive oscillometric central (aortic) BP and oscillometric brachial BP in normal-weight, overweight and obese female patients**

|  | **Total** | **Normal-weight** | **Overweight** | **Obesity** | ***P* value** |
| --- | --- | --- | --- | --- | --- |
| Patients, n | 47 | 9 | 21 | 17 |  |
| **Invasive central (aortic) BP** | | | | |  |
| SBP (mmHg) | 139.6 ± 24.1*  (140.5) | 139.1 ± 21.1  (133.3) | 139.5 ± 28.8  (143.4) | 139.9 ± 20.3*  (136.1) | 0.99^#^ |
| DBP (mmHg) | 64.7 ± 9.2**  (65.8) | 67.1 ± 10.5  (69.0) | 62.9 ± 9.9**  (65.8) | 65.6 ± 7.7  (65.5) | 0.47^#^ |
| MAP (mmHg) | 94.7 ± 12.9  (94.7) | 97.1 ± 15.1  (95.3) | 92.9 ± 14.6  (94.7) | 95.6 ± 9.3  (93.9) | 0.68^#^ |
| PP (mmHg) | 74.9 ± 22.1***  (75.8) | 72.0 ± 12.9  (73.0) | 76.6 ± 25.9*  (77.0) | 74.3 ± 21.5*  (75.3) | 0.87^#^ |
| **Non-invasive oscillometric central (aortic) BP** | | | | |  |
| SBP (mmHg) | 137.9 ± 24.2  (137.6) | 136.4 ± 17.9  (137.6) | 136.6 ± 29.4  (136.9) | 140.6 ± 20.9*  (143.1) | 0.87^#^ |
| DBP (mmHg) | 67.5 ± 9.4***  (69.0) | 71.9 ± 9.8  (70.3) | 65.5 ± 9.4***  (66.8) | 67.6 ± 8.9  (66.3) | 0.47^#^ |
| MAP (mmHg) | 94.1 ± 12.6  (93.9) | 97.4 ± 11.8  (96.5) | 92.0 ± 15.1  (90.6) | 95.1 ± 9.3  (95.0) | 0.54^#^ |
| PP (mmHg) | 70.5 ± 22.9***  (68.8) | 64.5 ± 16.5  (67.3) | 71.1 ± 25.5*  (66.6) | 72.9 ± 22.9***  (75.4) | 0.67^#^ |
| **Oscillometric brachial BP** | | | | | |
| SBP (mmHg) | 142.4 ± 21.3  (140.0) | 143.1 ± 16.0  (138.0) | 139.8 ± 26.3  (139.0) | 145.4 ± 17.1  (145.0) | 0.73^#^ |
| DBP (mmHg) | 78.2 ± 8.4  (78.0) | 80.3 ± 9.2  (77.0) | 76.1 ± 8.9**  (77.0) | 79.7 ± 7.2  (81.0) | 0.30^#^ |
| MAP (mmHg) | 101.6 ± 14.6  (101.0) | 99.3 ± 12.7  (98.0) | 99.8 ± 17.6  (101.0) | 105.1 ± 11.2  (106.0) | 0.49^#^ |
| PP (mmHg) | 64.2 ± 17.6**  (65.0) | 62.8 ± 10.6  (65.0) | 63.7 ± 21.4  (63.0) | 65.7 ± 16.1*  (71.0) | 0.91^#^ |

*Data are mean ± SD (Median). Normal weight, BMI: 18.5-24.9 kg/m^2^; Overweight, BMI: 25.0-29.9 kg/m^2^; Obesity, BMI: ≥30 kg/m^2^. BMI, body mass index; BP, blood pressure; SBP, systolic blood pressure; DBP, diastolic blood pressure; MAP, mean arterial pressure; PP, pulse pressure; P value: ^#^one-way analysis of variance. Sex comparison between the total group and the groups with the same BMI was performed using Student's t-test.*

**P<0.05, comparison with male group (Table in S1 Table)*

***P<0.01, comparison with male group (Table in S1 Table)*

****P<0.001, comparison with male group (Table in S1 Table)*
