# Supplementary material for: Invasively measured and estimated central blood pressure using the oscillometric algorithm Antares in patients with and without obesity
Source: PLoS One. 2023 Dec 14;18(12):e0294075. doi: 10.1371/journal.pone.0294075 (PMC10721029; doi:10.1371/journal.pone.0294075)
Supplement: S4 Table — BMI, Body Mass Index; bSBP, brachial systolic blood pressure; bMAP, brachial mean arterial pressure; bDBP, brachial diastolic blood pressure; bPP, brachial pulse pressure; cSBP, central systolic blood pressure; cMAP, central mean arterial pressure; cDBP, central diastolic blood pressure; cPP, central pulse pressure. **P<0.001. (DOCX) [file pone.0294075.s004.docx]

**S4 Table. Partial correlation coefficients between non-invasive central (aortic) BP variables and brachial BP variables adjusted for age and sex**

|  | **BMI** | **bSBP** | **bMAP** | **bDBP** | **bPP** | **cSBP** | **cMAP** | **cDBP** | **cPP** |
| --- | --- | --- | --- | --- | --- | --- | --- | --- | --- |
| **BMI** |  |  |  |  |  |  |  |  |  |
| **bSBP** | 0.055 |  |  |  |  |  |  |  |  |
| **bMAP** | 0.066 | 0.944** |  |  |  |  |  |  |  |
| **bDBP** | 0.088 | 0.738** | 0.825** |  |  |  |  |  |  |
| **bPP** | 0.022 | 0.912** | 0.784** | 0.397** |  |  |  |  |  |
| **cSBP** | -0.001 | 0.964** | 0.932** | 0.683** | 0.897** |  |  |  |  |
| **cMAP** | 0.006 | 0.903** | 0.933** | 0.912** | 0.675** | 0.887** |  |  |  |
| **cDBP** | 0.042 | 0.624** | 0.706** | 0.930** | 0.285** | 0.547** | 0.852** |  |  |
| **cPP** | -0.024 | 0.804** | 0.722** | 0.303** | 0.910** | 0.889** | 0.588** | 0.102 |  |

BMI, Body Mass Index; BP, blood pressure; bSBP, brachial systolic blood pressure; bMAP, brachial mean arterial pressure; bDBP, brachial diastolic blood pressure; bPP, brachial pulse pressure; cSBP, central systolic blood pressure; cMAP, central mean arterial pressure; cDBP, central diastolic blood pressure; cPP, central pulse pressure.

***P*<0.001
