# Supplementary figures and images for: Invasively measured and estimated central blood pressure using the oscillometric algorithm Antares in patients with and without obesity
Source: PLoS One. 2023 Dec 14;18(12):e0294075. doi: 10.1371/journal.pone.0294075 (PMC10721029; doi:10.1371/journal.pone.0294075)

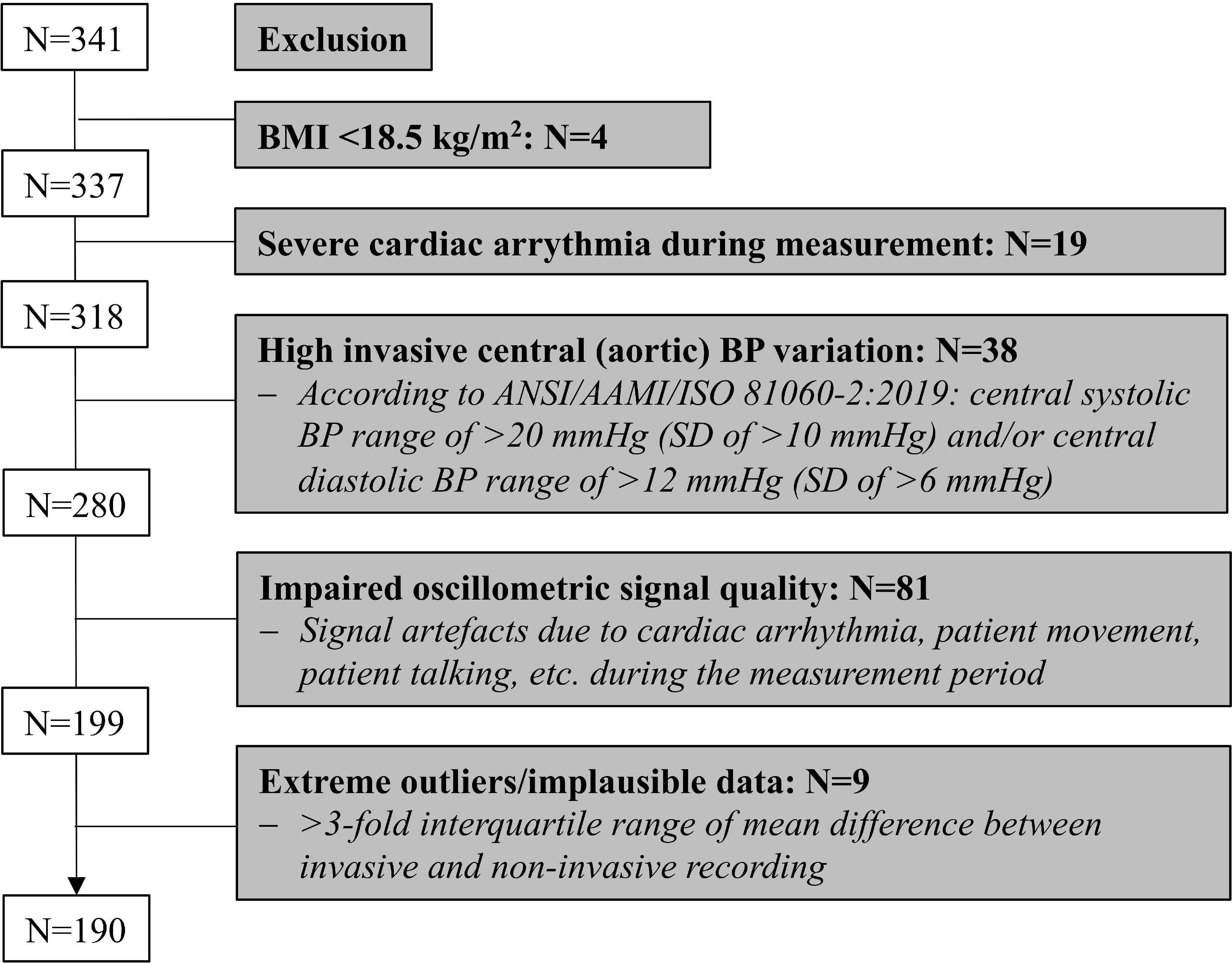

Supplement: S1 Fig — BMI, body mass index; BP, blood pressure; SD, standard deviation. (TIF) [file pone.0294075.s005.tif]
